# NCHD handover in the acute mental health setting: a quality improvement initiative implementing an electronic handover tool

**DOI:** 10.1136/bmjoq-2024-002978

**Published:** 2025-01-06

**Authors:** Stelios Naxakis, Megan Wafer, Syeda Gardezi, Aamina Sadia, Rimsha Mujahid, Karen O’Connor

**Affiliations:** 1 Psychiatry, University College Cork, Cork, Ireland; 2 University College Cork, Cork, Ireland

**Keywords:** Mental health, Communication, Continuity of Patient Care, Continuous quality improvement, Patient Handoff

## Abstract

**Background:**

Cork University Hospital, Ireland’s largest teaching hospital, faced challenges in maintaining consistent handover processes in its Acute Mental Health Unit (AMHU). Prior to 2019, handovers relied on informal methods, risking information loss and compromising patient care. This quality improvement (QI) initiative aimed to standardise handover practices using an electronic tool integrated with the ISBAR communication protocol.

**Objectives:**

The project aimed to ensure accurate clinical information recording, improve patient care and safety, centralise handover material, enhance clinical transparency and accountability, and measure handover quality using the electronic tool.

**Methods:**

Using a Plan-Do-Study-Act (PDSA) model, the initiative began with a critical incident in July 2020, prompting the creation and piloting of an electronic ‘handover tool’ aligned with ISBAR. Subsequent PDSA cycles included mandatory policy implementation and educational interventions to reinforce tool usage and adherence to communication standards.

**Results:**

The electronic handover tool improved handover practices, with increased compliance to recommended criteria and enhanced tool utilisation. Notable improvements followed targeted educational interventions, leading to more comprehensive and standardised handover entries. These improvements enhanced communication and information transfer among NCHDs, contributing to better continuity of care and patient safety.

**Conclusions:**

The QI initiative successfully standardised handover processes and improved communication among NCHDs in the AMHU. While improvements were observed, ongoing efforts are needed to address challenges and sustain effectiveness. Continuous training, feedback mechanisms and further refinement of the handover tool are essential for long-term success. Future directions include exploring additional technological solutions and reinforcing a culture of effective communication.

**WHAT IS ALREADY KNOWN ON THIS TOPIC**

- Handover is crucial for patient safety, with 25%–40% of adverse incidents linked to communication failures. In psychiatry, handovers are particularly complex due to the interdisciplinary nature of care and the range of patient issues. The ISBAR framework is widely adopted to address these challenges by standardising communication.

**WHAT THIS STUDY ADDS**

- This study shows that the introduction of an electronic handover tool, integrated with ISBAR, significantly improves communication among doctors in the psychiatric unit. Compliance with handover standards increased, improving patient safety and continuity of care.

**HOW THIS STUDY MIGHT AFFECT RESEARCH, PRACTICE OR POLICY**

- The success of the electronic handover tool in psychiatry suggests it could be a model for broader implementation across other medical settings. The ISBAR framework, combined with ongoing training and technology, enhances handover efficiency, which is crucial for reducing errors and improving patient care.

## Problem

 Cork University Hospital (CUH) stands out as Ireland’s largest teaching hospital, boasting a unique status as the country’s sole model 4 (specialist academic teaching) hospital, where all acute medical and surgical specialities converge on a single campus. This centralised setup contributes to its reputation as one of the busiest healthcare facilities nationwide, catering to local needs and supra-regional services to the southern third of Ireland.

At CUH, psychiatric handovers occur at strategic intervals throughout the week to ensure seamless transitions in patient care. During weekdays, these handovers happen four times a day, covering various shifts from morning to night. On weekends, the frequency reduces to two times per day. The intricate nature of these handovers, involving up to four doctors within 24 hours, underscores the importance of a robust protocol to prevent information loss and oversight of critical tasks.^1^

CUH encountered significant hurdles in maintaining consistent and accountable handover processes. Before the introduction of an electronic handover tool in 2019, information exchange relied heavily on informal methods, lacking documented evidence or standardised procedures. This informal approach not only posed risks of information loss but also compromised continuity of care, as evidenced by a critical incident involving a patient who did not receive a timely medical review over a weekend, resulting in severe consequences.

Meanwhile, within the Acute Mental Health Unit (AMHU) at CUH, a specialised ward attends to older adults under psychiatry of later life. Due to the diverse medical needs of this demographic, junior doctors often address a wide range of concerns beyond mental health, especially during unscheduled shifts. Consequently, effective handovers require thorough discussions covering both psychiatric and medical aspects, underscoring the crucial role of clear communication in ensuring optimal patient care.

The introduction of an electronic handover tool marked a significant improvement in CUH’s handover practices. This tool, integrated with the ISBAR communication protocol, provided a structured framework for conveying patient information and facilitating face-to-face handovers between incoming and outgoing doctors.^2 3^ Moreover, they serve as valuable archives of past handover information, ensuring accessibility and accountability in patient care.

The significance of effective handover processes cannot be overstated, as evidenced by their impact on patient safety and outcomes. Studies and quality improvement (QI) initiatives, both locally and internationally, have emphasised the importance of structured communication frameworks, such as ISBAR, in enhancing patient care and reducing errors.^1^,^9^ CUH’s adoption of the electronic handover tool aligns with these findings, reflecting a commitment to continuous improvement and optimal patient care.

## Introduction

Ineffective handover processes contribute significantly to adverse patient outcomes, accounting for 25%–40% of negative incidents according to a European Commission project.^2 3^

Handover, as delineated by the National Patient Safety Agency in 2004, signifies the transfer of professional responsibility and accountability for specific aspects of patient care, whether temporary or permanent.^4^

Despite the limited literature on doctor-to-doctor handover within psychiatry, notable QI initiatives led by Till *et al*, Skelton *et al* and Lazzari sought to implement electronic handover systems. Their findings underscored the transformative impact of formalising out-of-hours handover processes. By reducing errors, enhancing clarity and promoting seamless communication, the adoption of electronic handover systems led to tangible improvements in patient care quality.^2 3 7^

Furthermore, a study conducted by Acharya *et al* in 2017 demonstrated the efficacy of simulation-based educational sessions focused on the SBAR tool; psychiatric trainees exhibited significant improvements in confidence, skills, and the clinical application of the SBAR framework.^5^ The study highlighted the potential of structured training sessions to enhance patient care by instilling structure, confidence and empowerment in handover interactions among medical professionals.^5^

The ISBAR communication framework has emerged as a cornerstone for facilitating clear and concise communication in hospital settings. Originally developed by the US Navy and subsequently endorsed by the WHO, ISBAR ensures structured communication by outlining key elements: identification, situation, background, assessment and recommendation.^6 7^ This framework has garnered particular attention in psychiatric settings, where the complexity of patient care often requires doctors to manage unscheduled cases across broad geographical areas and multiple sites, presenting unique challenges.^3^,^8^

## Aims & objectives

The electronic handover tool QI project aimed to standardise doctor-to-doctor handover using the ISBAR communication format. Its primary objective was to develop a system that facilitates the recording of accurate and high-quality clinical information, thereby ensuring effective patient care and safety.

The project had specific aims, including:

- Centralising handover material for accessibility by staff on any registered HSE desktop/laptop.
- Enhancing clinical transparency and accountability for handed-over tasks, thereby facilitating incident reviews where handover was implicated.
- Measure the quality of doctor-to-doctor handover through assessment of the handover tool.

## Design

The implementation of a Plan-Do-Study-Act (PDSA) QI model guided this project. Initially led by a psychiatric trainee with support from a liaison psychiatry consultant, later joined by the clinical director of the AMHU at CUH, the project aimed to enhance handover processes.

Due to the absence of baseline measurements resulting from the lack of a standardised or documented handover process, the project’s first PDSA cycle was prompted by a significant critical incident.

### PDSA cycle 1

#### Plan

Led by a psychiatric trainee with support from a liaison psychiatry consultant, the project aimed to enhance handover processes at the AMHU, CUH. In July 2020, an adverse clinical outcome prompted an educational/tutorial intervention to be offered to all NCHDs. The session aimed to discuss handover importance and propose methods to improve the quality of handover, including the introduction of a formal handover document.

#### Do

The tutorial session provided a platform for discussion and idea generation regarding handover improvement. Despite initial concerns from doctors about potential increased administrative duties, informal feedback from the session led to a decision to pilot a proposed handover document. Criteria for compliance with the document were developed (table 1), and an electronic ‘handover tool’ was created.

**Table 1:** Identifying recommended criteria for handover as per ISBAR framework

Recommendation criteria	Handover tool ISBAR
1.1	Included date of handover as well as named person providing handover and receiving handover
1.2	I—At least three patient identifiers present (ie, name, date of birth, medical records number)
1.3	S—Situation clearly and adequately provides information relating to the clinical handover
1.4	B—Background information is clear and adequate to the details of clinical handover
1.5	A—Assessment information is clear and adequate to the details of clinical handover
1.6	R—Recommendations are clear and actionable by receiver of handover

This tool, aligned with the ISBAR mnemonic, featured fillable boxes to ensure comprehensive handover information (online supplemental appendix—figure 2). It was saved in the South Lee Mental Health Service shared folder, with a corresponding standard operating procedure.

#### Study

Feedback from users of the handover tool during the pilot phase was collected and analysed. Observations focused on the ease of use, completeness of information entered, and any perceived impact on handover efficiency and patient care quality. The effectiveness of the tool in standardising handover procedures and improving communication among medical staff was carefully evaluated.

#### Act

Based on the feedback and observations from the pilot phase, adjustments were made to enhance the utilisation of the handover tool and the accompanying standard operating procedure. Modifications aimed to improve user experience, address usability concerns and streamline handover processes. Additional training and support were provided to ensure all staff members were proficient in using the tool effectively. The revised handover tool and updated procedures were then implemented as standard practice within the AMHU at CUH. Ongoing monitoring and evaluation were conducted to identify further areas for improvement and ensure the sustained effectiveness of the handover protocol.

### PDSA cycle 2

#### Plan

In response to the significant change in the weekend on-call shift schedule for NCHDs at the AMHU in July 2022, the clinical director recognised the increased risk of negative clinical outcomes resulting from handover issues. It was planned to implement a mandatory policy for the use of the NCHD handover tool to mitigate these risks. This policy aimed to address the doubling of doctor-to-doctor handovers during weekends by standardising handover procedures and improving communication.

#### Do

The mandatory policy for the use of the NCHD handover tool was implemented, requiring all NCHDs to use the tool for handovers during their shifts. Unlike previous orientations, the orientation for upcoming NCHDs placed a stronger emphasis on the mandatory use of the handover tool, adopting a top-down approach. NCHDs were instructed on the correct utilisation of the tool and the parameters of the ISBAR communication system. The policy was put into practice immediately, with NCHDs instructed to use the handover tool for all handovers during their shifts.

#### Study

Feedback and compliance data regarding the use of the handover tool were collected and analysed following the implementation of the mandatory policy. Observations were made regarding the frequency and quality of handovers, as well as any perceived improvements in communication. Compliance rates with the policy were monitored closely to assess the effectiveness of the intervention.

#### Act

Based on the feedback and compliance data collected during the study phase, adjustments were made to further improve the effectiveness of the mandatory policy for handover tool usage. Additional training and support were provided to NCHDs to address any challenges or barriers to compliance. The importance of using the handover tool for all handovers was reinforced through ongoing communication and feedback mechanisms.

### PDSA cycle 3

#### Plan

In September 2023, an educational intervention was planned for NCHDs in the AMHU, South Lee Mental Health Service, focusing on the utilisation of the handover tool. The session aimed to highlight compliance rates over the past 3 years (September 2021, September 2022 and September 2023) and share data regarding adherence to ISBAR communication standards within the handover tool.

#### Do

The educational intervention was conducted with a focus on both reinforcing the use of the handover tool and providing NCHD’s with constructive feedback on their current performance. The session aimed to communicate the current levels of compliance with the tool, emphasising where improvements were needed and where good practices were already in place. Visual aids were used to clearly highlight areas of success—where NCHD’s were effectively using the tool—and areas requiring improvement, enduring that NCHD’s could easily identify the gaps in compliance. The intervention reiterated the critical role of high-quality clinical handovers, emphasising the risks involved when communication is unclear or incomplete. Real-life examples, witnessed within the NCHD group were used to illustrate both effective and ineffective handover practices. These examples were drawn from recent handovers within the AMHU, making the feedback directly relevant and relatable to NCHDs. The session aimed to foster buy-in from NCHDs by acknowledging the challenges faced and encouraging them to take ownership of the handover process. It highlighted the direct impact that their diligence in handovers could have on patient care, safety and outcomes. NCHDs were informed that their efforts would be reassessed in the coming months, ensuring that the improvements in handover would be continuously monitored.

#### Study

Following the educational intervention, a re-evaluation of the handover tool was conducted in December 2023 to assess any changes or improvements resulting from the intervention. Compliance rates with the use of the handover tool and adherence to ISBAR communication standards were compared with previous data to determine the impact of the educational intervention on handover practices.

#### Act

Based on the findings from the re-evaluation conducted in December 2023, adjustments were made to further enhance the utilisation of the handover tool and adherence to ISBAR communication standards. Additional training or support measures were implemented as needed to address any identified areas for improvement. Positive trends or improvements resulting from the educational intervention were reinforced, and ongoing efforts were made to sustain and build on these gains in handover quality and communication effectiveness.

## Results

The assessment of the handover tool aimed to gauge compliance with recommended handover criteria (table 1) and its utilisation during NCHD changeovers. NCHD handovers took place daily at 09:00 in the morning and 17:00 (16:00 on Fridays) during weekdays, with weekend schedules set for 09:00 and 21:00. Handover occurrences were categorised as either morning, evening or both sessions based on their presence at the specified times, along with tracking the monthly entries for handover.

Following the initial implementation of the handover tool, compliance with recommendations 1.1, 1.2 and 1.6 remained notably low, with rates of 11%, 11% and 16%, respectively. These recommendations emphasise documenting the NCHDs involved in the handover, the date of handover, patient identification and detailing requested tasks. Compliance with recommendation 1.3 stood at 29%, focusing on providing a clear and adequate description of the situation at hand. Conversely, compliance with recommendations 1.4 and 1.5 showed positive outcomes, with rates of 84% and 99%, respectively, indicating a clear and adequate description of patient background information and the NCHD’s assessment of the case.(table 2)

**Table 2:** - Representation of measured compliance with recommended critera for handover from 2020 to 2023

	1.1 (%)	1.2 (%)	1.3 (%)	1.4 (%)	1.5 (%)	1.6 (%)
September 2020	11	11	29	84	99	14
September 2021	22	12	31	93	100	14
September 2022*	61	11	31	86	95	17
September 2023	71	15	30	93	99	12
December 2023†	70	92	61	90	100	38

* Following PDSA cycle 2

† Following PDSA cycle 3

From September 2021 to September 2023, trends in compliance remained relatively stable. Compliance with recommendations 1.2 (11%–15%), 1.3 (30%–31%) and 1.6 (12%–17%) continued to be low, while compliance with recommendations 1.4 (86%–93%) and 1.5 (95%–100%) remained high. However, there was an upward trend in compliance with recommendation 1.1, increasing from 22% in September 2021 to 71% in September 2023 (table 2).

The above data suggested that NCHDs while continuing to appropriately communicate the background of the clinical scenario as well as their assessment of same, struggled with identifying patients (three identifiers or more), describing the situation pertaining to the case in question and developing clear and actionable tasks for handover. The data does suggest, however, that NCHDs became more proficient in identifying the date of handover as well as naming those involved in the handover process.

After PDSA cycle 2, which entailed the top-down implementation of the handover tool, there was not a significant shift in compliance with the recommended handover standards, except for criteria 1.1, which increased to 61% from 22% in the previous year (table 2).

Following PDSA cycle 3, featuring an educational session targeting areas for improvement with the same NCHD cohort evaluated in September 2023, there was a notable uptick in compliance with recommendations 1.2, 1.3 and 1.6. However, there was a slight decrease in compliance with criteria 1.1 and 1.4 (table 2).

In addition to evaluating compliance with recommended handover standards, an assessment of the specific utilisation of the handover tool was conducted. Data spanning September 2021, 2022 and 2023, along with December 2023 following PDSA cycle 3, was analysed(table 3). Initially, the utilisation of the handover tool was scant, with only 17% utilisation during morning handover sessions and a moderate 70% during evening sessions. Notably, there were no instances of concurrent tool use in both morning and evening periods in September 2021. Overall, the monthly use of the handover tool was poor, with merely eight documented handover entries.

**Table 3:** Representation of the overall use of the electronic handover tool from 2021 to 2023

	September 2021	September 2022*	September 2023	December 2023†
AM recorded	17%	37%	37%	60%
PM recorded	70%	73%	47%	73%
Both (AM and PM) recorded	0%	30%	13%	50%
Total entries	8	70	41	101

* Following PDSA cycle 2.

† Following PDSA cycle 3.

Following PDSA cycle 2 in September 2022, there was an improvement in morning handover tool utilisation to 37%, while evening utilisation remained steady at 73% compared with the previous year. There was a notable increase of 30% in instances of tool usage during both morning and evening handover periods, compared with none in the previous year. The total number of entries in the handover tool spiked to 70 in September 2022, a significant rise from the mere eight entries in September 2021.(table 3)

In September 2023, before PDSA cycle 3, the utilisation of the handover tool during the morning handover period remained steady at 37%. However, there was a notable decline of 26% in the tool’s usage during the evening handover period, along with a 17% decrease in instances where the tool was employed for both morning and evening handovers. During this month, there were a total of 41 entries recorded, a decrease from the previous year’s tally of 70 entries (table 3).

Following PDSA cycle 3, a significant increase in handover tool utilisation was observed for both morning and evening handover periods. Notably, half of the observed days showed the tool’s usage in both morning and evening sessions. Moreover, the monthly number of handover entries increased to 101, signifying a substantial improvement from the 41 entries recorded before PDSA cycle 3 (table 3).

## Limitations

The interpretation of the presented data should acknowledge the complex and variable nature of clinical care and staff dynamics. Clinical handover processes are influenced by the unique caseload of each day, which has a direct impact on the utilisation of the handover tool, thus fluctuations in handover tool usage should be viewed in the context of this inherent variability.

It is important to recognise that the reported data is subject to random variation and potential selection bias; the NCHDs engaging in the handover process were non-similar at each investigated interval, except for September 2023 and December 2023 following PDSA cycle 3. This variability in personnel may influence compliance rates, as NCHDs differed in their familiarity with and adherence to the handover tool. Moreover, the Hawthorne effect may introduce bias into the observed data, particularly following PDSA cycle 3. The NCHDs were aware of being monitored for their engagement with the handover tool, which could have influenced their behaviour and compliance levels.

NCHD orientation took place every July and compliance and use of the handover tool were measured 2 months post-orientation. A 2-month follow-up assessment was chosen arbitrarily and may not capture potential fluctuations in compliance over shorter or longer periods. Therefore, the findings should be interpreted with caution regarding the timing of assessment and intervention as well as the potential impact on observed trends.

## Discussion

The QI project, focused on enhancing handover processes within the AMHU at CUH, provides valuable insight into the effectiveness of interventions aimed at improving communication and standardising procedures among NCHDs. Trends observed throughout the PDSA cycles highlight the following themes: compliance with recommended handover standards, utilisation of the handover tool, the impact of educational interventions, and challenges faced with opportunities for sustained improvements.

As detailed in online supplemental appendix 1, the identified change ideas are linked to specific drivers that support the project’s overall aim of standardising NCHD handovers to enhance patient safety, communication, and continuity of care.

The primary drivers include:

- Effective and structured communication, emphasising the importance of using a standardised ISBAR format and ensuring clear patient identification and comprehensive handover details.
- Compliance and adoption of the electronic handover tool, which focuses on providing clear guidance on the tool’s usage, designing user-friendly interfaces, and ensuring accessibility across HSE-registered devices.
- Training and education, aimed at reinforcing the use of the handover tool through regular educational sessions, simulation-based training, and induction for new NCHDs.
- Policy enforcement and accountability, ensuring that mandatory policies are in place for tool usage, and monitoring compliance with job descriptions and handover tasks.
- Ongoing monitoring and feedback, which focuses on gathering continuous feedback, conducting audits and using PDSA cycles to sustain improvements.

These change ideas, which are visually represented in the driver diagram (online supplemental appendix 1), demonstrate a structured approach to addressing key challenges and improving handover practices. By aligning specific interventions with each driver, the project facilitates a more effective handover process and better communication among NCHDs.

### Compliance with recommended handover standards

The data shows a mixed level of compliance with the recommended handover criteria outlined in table 1. While some criteria, such as providing clear background information and clinical assessments, showed consistently high compliance rates, others, such as including patient identifiers and developing clear tasks for handover, remained challenging for NCHDs.

Notably, there was an improvement in compliance with recommendation 1.1 (documenting date and personnel involved) following the top-down implementation of the handover tool, indicating the effectiveness of local policy enforcement in certain aspects. However, sustained improvements across all criteria were not observed consistently, suggesting ongoing challenges in certain areas of handover communication despite the above-outlined interventions.

### Utilisation of the handover tool

The utilisation of the handover tool showed an upward trend across the PDSA cycles, with significant improvements observed following each intervention. Initially, the tool’s usage was limited, but it gradually increased, particularly after PDSA cycle 2 and cycle 3 interventions. The introduction of mandatory policies and educational sessions contributed to enhanced tool adoption among NCHDs.

Notably, the increase in tool utilisation was accompanied by a rise in the number of documented handover entries, suggesting a positive correlation between tool usage and comprehensive handover practices.

### Impact of educational interventions

The educational sessions implemented in PDSA cycle 3 resulted in notable improvements in compliance with certain handover criteria, particularly in areas where previous interventions had not yielded significant changes. The educational intervention emphasised that the success of the project relied on the active participation of the NCHD group, and that their contributions would directly influence the quality of care delivered within the unit. The combination of feedback, real-world examples outlined in PDSA cycle 3, as well as continuous monitoring of progress was aimed at building both competence and confidence among NCHDs, ultimately fostering a culture of effective handovers and communication.

However, it is essential to note that despite improvements in some criteria, there were slight decreases in compliance with others. This suggests the need for targeted interventions and continuous reinforcement of best practices to address specific challenges comprehensively.

### Challenges and opportunities for improvement

The data highlights persistent challenges in certain aspects of handover communication, such as identifying patients with sufficient identifiers and developing clear tasks for handover.

While interventions led to improvements, sustained compliance across all recommended criteria remained a challenge. This underscores the complexity of behavioural change and the need for ongoing support and reinforcement.

Opportunities for further improvement include targeted training and support initiatives tailored to address the standard of communication regarding patient handover, both initially during orientation, as well as continuously at regular intervals.

### Sustainability and future directions

Prolonged success in improving handover processes relies on sustainability efforts beyond the initial interventions, as preliminarily suggestive of the data collected. Ongoing monitoring, feedback mechanisms and regular training sessions will be crucial for maintaining compliance and fostering a culture of effective communication.^9^

Future directions may include exploring technological solutions or refining the existing handover tools to enhance usability and address specific challenges identified in handover practices.

## Conclusion

In conclusion, the results of the QI project demonstrate progress in enhancing handover processes within the AMHU. While interventions have improved compliance and tool utilisation, ongoing efforts are needed to address persistent challenges and encourage sustained effectiveness.

The insights from this QI project resonate with findings from Fryman *et al*’s study on ‘Quality Improvement Approach to Standardization and Sustainability of the Hand-off Process’ (2017); initially, poor compliance with handover was observed 6 months post implementation, which improved following targeted interventions such as direct observation with feedback.^10^

Future transformative and collaborative endeavours are required of leaders and changemakers regarding continued patient care and communication improvement. Tools and tactics such as frequent educational interventions, visual performance aids and positive critical feedback may prove crucial in driving sustainable positive change.

## supplementary material

10.1136/bmjoq-2024-002978 online supplemental file 1

## Data Availability

Data are available in a public, open access repository.
