# Supplementary material for: NCHD handover in the acute mental health setting: a quality improvement initiative implementing an electronic handover tool
Source: BMJ Open Qual. 2025 Jan 6;14(1):e002978. doi: 10.1136/bmjoq-2024-002978 (PMC11751932; doi:10.1136/bmjoq-2024-002978)

Appendix

1. Figure 1 - Drivers Diagram

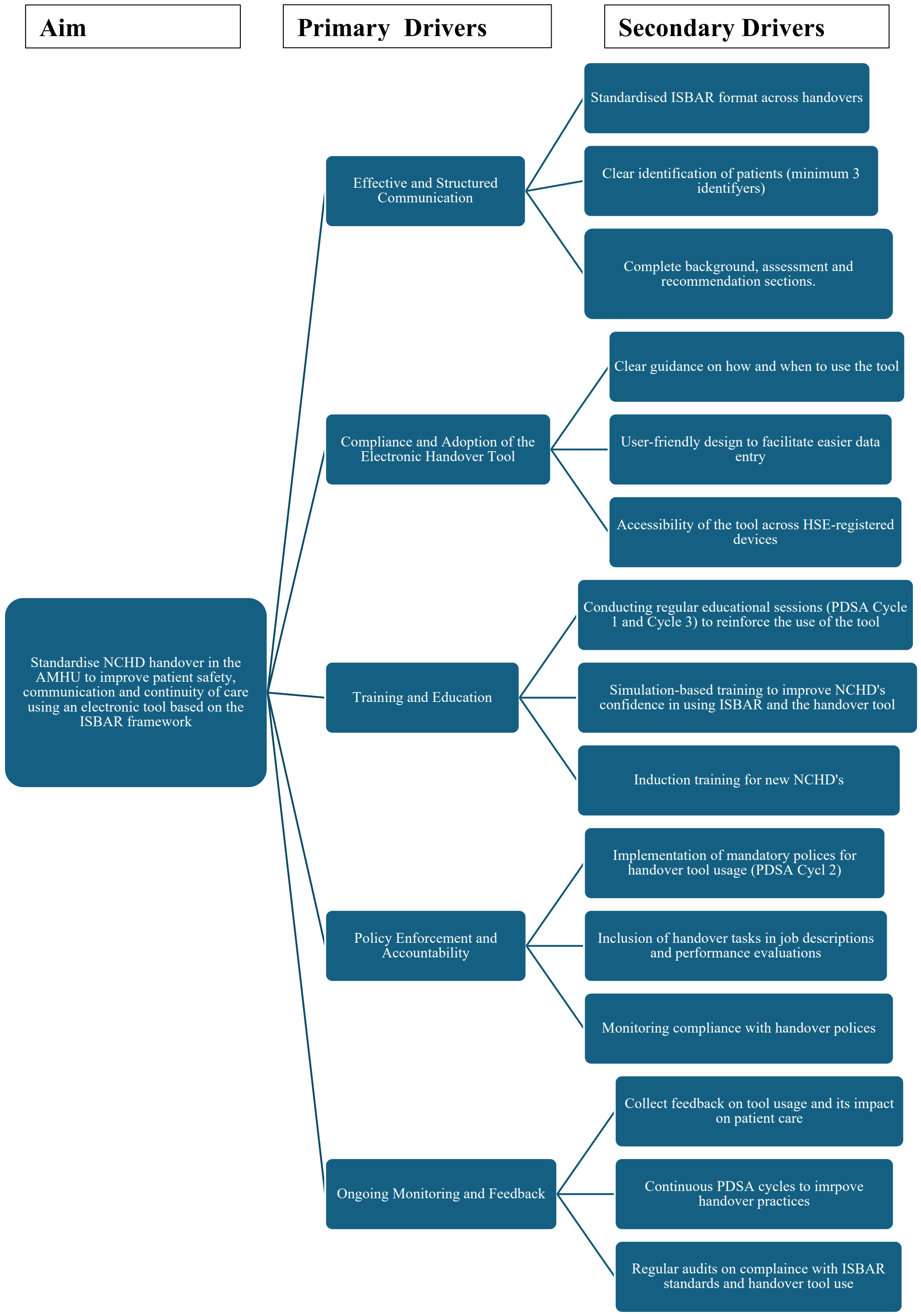

2. Figure 2 – Electronic Handover Tool Template

**Date**<br><br>**Time**<br><br>**From**<br><br>**To**	**I**<br>**(Patient Information)**	**S**<br>**(Situation)**	**B**<br>**(Brief History)**	**A**<br>**(Clinical Assesment)**	**R**<br>**(Task to Handover)**	**Task completed?**<br><br>**YES/NO**

3. Figure 3 – Run Chart showing compliance with Handover Recommendations (2020 – 2023)

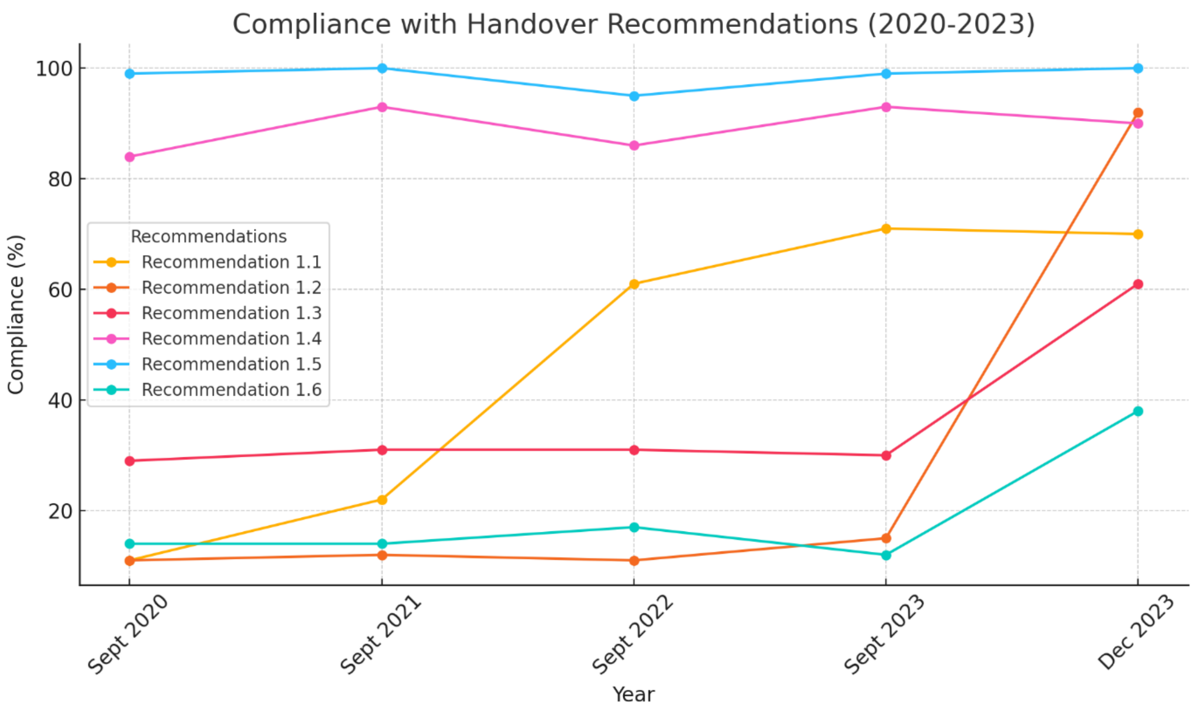

Supplement: online supplemental file 1 [file bmjoq-14-1-s001.pdf]
